# Hemodynamic and Metabolic Tolerance of Acetate-Free Biofiltration in Mechanically Ventilated Critically Ill Patients: A Real-Life Study

**DOI:** 10.3390/jcm10245729

**Published:** 2021-12-07

**Authors:** Anna Gouin, Pierre Tailpied, Olivier Marion, Laurence Lavayssiere, Chloé Medrano, Marie-Béatrice Nogier, Bruno Seigneuric, Nassim Kamar, Olivier Cointault, Stanislas Faguer

**Affiliations:** 1 Department of Nephrology and Organ Transplantation, Intensive Care Unit, University Hospital of Toulouse, F-31000 Toulouse, France; gouin.a@chu-toulouse.fr (A.G.); pierre.tailpied@gmail.com (P.T.); marion.o@chu-toulouse.fr (O.M.); lavayssiere.l@chu-toulouse.fr (L.L.); medrano.c@chu-toulouse.fr (C.M.); nogier.mb@chu-toulouse.fr (M.-B.N.); seigneuric.b@chu-toulouse.fr (B.S.); kamar.n@chu-toulouse.fr (N.K.); cointault.o@chu-toulouse.fr (O.C.); 2 Faculty of Medicine, University Paul Sabatier, F-31000 Toulouse, France; 3 National Institute of Health and Medical Research, UMR 1297, Institute of Metabolic and Cardiovascular Diseases, Rangueil Hospital, F-31000 Toulouse, France

**Keywords:** dialysis, acetate-free biofiltration, hemodynamic tolerance, CO_2_, mechanical ventilation

## Abstract

Intradialytic hypotension can lead to superimposed organ hypoperfusion and ultimately worsens long-term kidney outcomes in critically ill patients requiring kidney replacement therapy. Acetate-free biofiltration (AFB), an alternative technique to bicarbonate-based hemodialysis (B-IHD) that does not require dialysate acidification, may improve hemodynamic and metabolic tolerance of dialysis. In this study, we included 49 mechanically ventilated patients requiring 4 h dialysis (AFB sessions *n* = 66; B-IHD sessions *n* = 62). Whereas more AFB sessions were performed in patients at risk of hemodynamic intolerance, episodes of intradialytic hypotension were significantly less frequent during AFB compared to B-IHD, whatever the classification used (decrease in mean blood pressure ≥ 10 mmHg; systolic blood pressure decrease >20 mmHg or absolute value below 95 mmHg) and after adjustment on the use of vasoactive agent. Diastolic blood pressure readily increased throughout the dialysis session. The use of a bicarbonate zero dialysate allowed the removal of 113 ± 25 mL/min of CO_2_ by the hemofilter. After bicarbonate reinjection, the global CO_2_ load induced by AFB was +25 ± 6 compared to +80 ± 12 mL/min with B-IHD (*p* = 0.0002). Thus, notwithstanding the non-controlled design of this study, hemodynamic tolerance of AFB appears superior to B-IHD in mechanically ventilated patients. Its use as a platform for CO_2_ removal also warrants further research.

## 1. Introduction

Because intradialytic hypotension can lead to superimposed organ hypoperfusion and ultimately mitigate long-term kidney outcomes after acute kidney injury, it remains a concern in critically ill patients requiring kidney replacement therapy. Intradialytic hypotension results from an inadequate compensatory response (heart rate, myocardial contractility, vascular tone and splanchnic flow shifts) to ultrafiltration rate, cardiac output decrease and/or arterial tonus changes during the session [1].

Dialysate composition may influence hemodynamic tolerance of intermittent hemodialysis (IHD) performed in the ICU [2,3]. Usual IHD techniques use bicarbonate-rich dialysate (B-IHD) but require acidification of the dialysate with acetate, citrate or hydrochloric acid to avoid calcium carbonatation within the dialysis filter. Beyond the direct effect of acetate on cardiac contractility and vascular tone, dialysate acidification by itself may also lead to systemic vascular effects, including on the pulmonary vascular bed, due to the release of high amounts of carbon dioxide in the blood outflow, whatever the acid used [4]. Thus, there is an unmet need to optimize tolerance of IHD, especially in critically ill patients, and modulating dialysate may reduce the rate of intradialytic hypotension.

Acetate-free biofiltration (AFB) is an alternative technique of IHD based on a bicarbonate-free dialysate (thus without the need for dialysate acidification) with post-filter reinjection of sodium bicarbonate. Few studies suggested that AFB may be better tolerated than B-IHD in patients receiving chronic dialysis [5], but results in acute kidney injury remain elusive [6]. Whether the potential beneficial hemodynamic effects of AFB may translate to critically ill patients remains to be established. In addition, recent studies in rats and in sheep suggested that hemodialysis with bicarbonate-free dialysate may be used as a platform of carbon dioxide removal to treat hypercapnic acidosis [7,8].

This report describes our findings of hemodynamic and metabolic outcomes in mechanically ventilated patients receiving dialysis with AFB or B-IHD.

## 2. Methods

Between February 2020 and March 2021, we prospectively included patients admitted to the ICU and receiving mechanical ventilation, who had intermittent sessions of hemodialysis.

B-IHD and AFB were performed with the Citrasate (Hemotech^®^, Toulouse, France) and Safebag (Hospal^®^, Meyzieu, France) dialysates, respectively. Sodium conductivity was 13.5 to 13.7 mSv. Membranes were the Elisio 21H (Nipro^®^, Cournon, France) or Evodial (Baxter^®^, Guyancourt, France) polyethersulfone filters. AFB dialysate composition was the following: sodium 140 mmol/L, chloride 146 mmol/L, calcium 1.5 mmol/L, magnesium 0.5 mmol/L, glucose 5.5 mmol/L, potassium 3.5 mmol/L (sodium conductivity was settled at 13.5 to 13.7 mS/cm). Solution of sodium bicarbonate that was reinjected had a concentration of 167 mmol/L. B-IHD dialysate composition was the following: sodium 140, chloride 108 mmol/L, potassium 3 mmol/L, calcium 1.65 mmol/L, magnesium 0.5 mmol/L, citrate 0.8 mmol/L, and glucose 1 g/L (sodium conductivity was settled at 14–14.5 mS/cm). Dialysate temperature was 1 °C below the patient temperature. Blood flow was settled 250 to 300 mL/min.

Characteristics of the patients were collected from their digitized medical charts. Blood pressures were measured using an arterial catheter. Intradialytic hypotension was diagnosed according to the KDOQI clinical practice guidelines (i.e., decrease in systolic blood pressure ≥ 20 mmHg or decrease in mean arterial pressure ≥ 10 mmHg) [9]. In addition, episodes of systolic blood pressure decrease below 95 mmHg were recorded. Hemodynamic parameters (systolic, diastolic and mean blood pressure, as well as heart rate and dose of norepinephrine) were collected each 30 min.

In a subset of AFB and B-IHD sessions, blood gases were measured before and after the filter for B-IHD, or before and after the filter and after the bicarbonate reinjection for AFB. The global CO_2_ load induced by dialysis sessions was approximated with the following formula: CO_2_ load = [total inflow CO_2_ content minus total outflow CO_2_ content] × blood flow × time.

Continuous variables were given as mean ± SEM and compared with the Mann–Whitney test. Discontinuous variables were given as number (percentages) and compared with Fisher’s exact test, after adjustment on the use of vasoactive agent at baseline. Hemodynamic parameters of AFB and B-IHD sessions were compared with a two-way ANOVA test with mixed-effects.

All patients included in this study gave informed consent. This study was conducted according to the Best Clinical Practices guidelines and the Declaration of Helsinki, as revised in 2004, and approved by the French national ethics committee (COOBRA study no. ID-RCB 2019-A03261-56).

## 3. Results

We included 49 mechanically ventilated patients (mean age 65 ± 12 years), who received 4 h sessions of AFB (*n* = 66) or B-IHD (*n* = 62) (Table 1). The mean volume of ultrafiltration prescribed was similar in both groups (1.8 ± 0.8 vs. 1.8 ± 0.9 L).

### 3.1. Hemodynamic Tolerance

**Figure 1:**
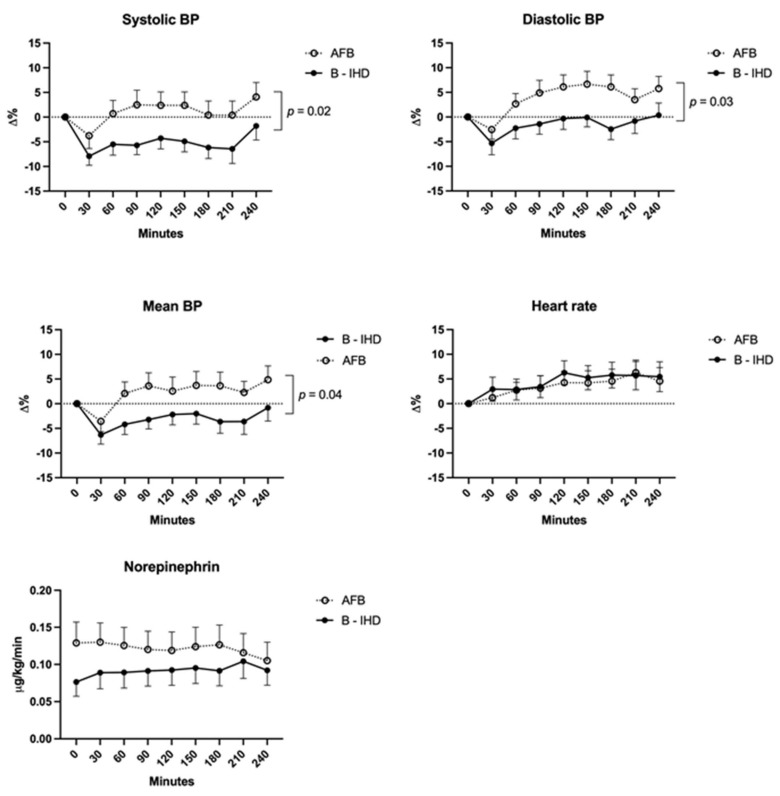
Hemodynamic monitoring during AFB (*n* = 66) and B—IHD (*n* = 62) sessions in 49 mechanically ventilated critically ill patients. AFB, acetate-free biofiltration; B-IHD, bicarbonate intermittent hemodialysis; BP, blood pressure; D%, percent change.

Whereas more AFB sessions were performed in patients of male gender (89 vs. 67%, *p* = 0.002), with underlying chronic obstructive broncho-pneumopathy (41 vs. 19%, *p* = 0.007), admitted for sepsis (70 vs. 48%, *p* = 0.02) or with ongoing vasoactive support (67 vs. 47%, *p* = 0.03), episodes of intradialytic hypotension were significantly less frequent during AFB compared to B-IHD, whatever the classification used (decrease in mean blood pressure ≥ 10 mmHg: 36/66 (54%) vs. 46/62 (74%) sessions, *p*-value = 0.02; systolic blood pressure decrease >20 mmHg: 27/66 (41%) vs. 40/62 (64%) sessions, *p*-value = 0.007). After the adjustment on the use of vasoactive agent at the start of the session, an episode of systolic blood pressure decrease below 95 mmHg was more frequent during dialysis sessions with B-IHD (25/62 (40%)) compared to AFB (16/66 (24%); adjusted *p*-value 0.03). Hemodynamic monitoring showed significantly better global tolerance of AFB sessions owing to increased diastolic blood pressure and stable systolic blood pressure, without concomitant increase in norepinephrine (Figure 1).

### 3.2. Carbon Dioxide Loading during Dialysis

In AFB sessions, optimal control of the acid-base status was obtained with a bicarbonate reinjection rate of 2.3 L/h with a blood flow of 250 mL/min (end dialysis: base excess 0.5 ± 2.6; chloride 104 ± 0.6 mmol/L), compared to 2.3 L/h and 300 mL/min, respectively (end dialysis: base excess −2.7 ± 4.6; chloride 106 ± 1.3 mmol/L).

**Figure 2:**
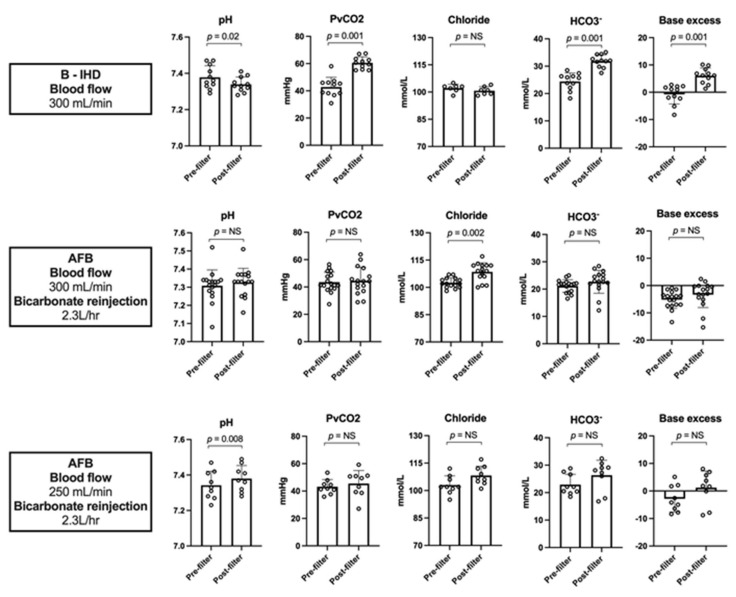
Venous gases in the blood returning to the patients during bicarbonate intermittent hemodialysis (B—IHD) and acetate-free biofiltration (AFB). PvCO_2_, venous carbon dioxide pressure; HCO_3_, bicarbonates.

Blood gases after the dialysis filter showed the respective metabolic impact of B-IHD and AFB: PvCO_2_, bicarbonate concentration and pH in blood returning to the patients were 60 ± 4.4 mmHg, 32 ± 2.3 mmol/L and 7.34 ± 0.04 compared to 45 ± 9.6 mmHg [*p* < 0.001], 24 ± 4.9 mmHg [pH < 0.001] and 7.35 ± 0.07 [*p* = 0.79], respectively (Figure 2).

Last, we estimated the net balance of CO_2_ induced by both techniques and the ability of dialysis with low-bicarbonate dialysate to be used as a platform for CO_2_ extraction. The blood exiting the AFB filter (before bicarbonate reinjection) was characterized by very low concentration of bicarbonate and partial pressure of CO_2_, leading to a decrease in the total CO_2_ content from 23 ± 3.3 to 5.2 ± 2.6 mmol/L (mean CO_2_ removal: 113 ± 25 mL/min). The global CO_2_ load (i.e., total CO_2_ content changes) induced by AFB sessions was +25 ± 6 compared to +80 ± 12 mL/min with B-IHD (Hodges-Lehmann difference −51.2 mL/min, *p* = 0.0002).

## 4. Discussion

In this study, we showed that hemodynamic tolerance was better in mechanically ventilated critically ill patients receiving AFB compared to conventional B-IHD, despite higher vasoactive support at the start of AFB sessions. This held true that the tolerance was assessed using the K/DOQI classification or the quantitative assessment of hemodynamic parameters. Because other dialysis and patient characteristics were balanced between the two groups or in favor of B-IHD, the discrepancies between the contents of the blood that returns to the patients during AFB and B-IHD probably account for the divergent hemodynamic tolerance. We first hypothesized that large amounts of CO_2_ in blood outflow during B-IHD may activate circulating inflammatory cells [10,11] and increase systemic inflammation with a subsequent decrease in the vascular tone. As an alternative hypothesis, the euhydric hypercapnic blood (i.e., high PvCO_2_, high bicarbonate levels, normal pH) that returns to the patient during B-IHD may modulate the vascular tone of the pulmonary artery [12,13,14] and subsequently alter the heart response during dialysis. These should be tested in further comparative studies in human or animal experiments.

Last, we confirmed in humans the potential of dialysis with low-bicarbonate dialysate to remove CO_2_ [7]. We first showed confirmed that the CO_2_ load was significantly reduced during AFB sessions compared to B-IHD sessions. This may be of utmost interest in patients at risk to develop hypercapnic acidosis (for instance, with chronic obstructive pulmonary disease or severe asthma) but also at risk of hypercapnia-induced high intracranial pressure. Second, we report a case of COVID-19-related acute respiratory distress syndrome complicated by refractory hypercapnia and core pulmonal successfully reversed by AFB. Even if the development of customized “balanced” dialysates with low chloride concentrations and no bicarbonate will be helpful in developing true “respiratory dialysis” [7], AFB may be already tested in cases of refractory hypercapnic conditions, as salvage therapy.

This study has some limitations, mainly related to its uncontrolled design. First, the risk factors of hemodialysis intolerance were not balanced between the two groups, but these risk factors were more frequent in the AFB sessions group. The positive results in favor of AFB we observed thus reinforce its probable superiority. Secondly, at baseline, hemodynamic status of patients was heterogeneous. Further comparative randomized studies are thus required to confirm our results, especially in patients with vasoactive support (i.e., with the highest risk to develop hypotension during sessions). Last, the mechanisms by which tolerance of AFB is superior to B-IHD still remain elusive and will require additional studies in humans or in animals.

## 5. Conclusions

We showed that AFB is safe in critically ill patients. Notwithstanding the non-controlled design of this study and its potential biases, our findings suggest that hemodynamic tolerance of AFB is superior to B-IHD in mechanically ventilated patients. If confirmed, it may reduce superimposed artificial kidney-induced kidney injuries and improve long-term kidney outcomes. Its use as a platform for CO_2_ removal also warrants further research.

## Figures and Tables

**Table 1:** Characteristics of patients and dialysis sessions. SD, standard deviation; ICU, intensive care unit; B-IHD, bicarbonate-based intermittent hemodialysis; AFB, acetate-free biofiltration.

Patients	*n* = 49		
Age (years; mean ± SD)	63 ± 13		
Male gender (*n*, %)	37 (75)		
Known heart disease (*n*, %) Chronic Kidney Replacement Therapy (*n*, %) SAPS2 score at the admission to the ICU	25 (51) 20 (40.8) 65 ± 18		
Cause of admission to the ICU Sepsis Acute respiratory distress syndrome Acute pulmonary oedema Cardiac surgery Hemorrhage Other	12 (24.5) 17 (34.7) 3 (6.1) 3 (6.1) 2 (2.1) 12 (24.5)		
**Characteristics of the Sessions**	**Dialysis Technique**		
	**B-IHD** ***n* = 62**	**AFB** ***n* = 66**	***p* Value**
Controlled-volume ventilation (*n*, %)	33 (55)	46 (70)	0.069
Vasopressive support (*n*, %) Dose of norepinephrine (μg/kg/min) At the start of the session (mean ± SD)	29 (47) 0.08 ± 0.16	44 (67) 0.12 ± 0.21	0.023 0.124
Volume expansion in the 6 preceding hours (*n*, %)	3 (4)	6 (9)	0.49
Blood pressure (mmHg) Mean (mean ± SD) Systolic (mean ± SD) Diastolic (mean ± SD)	85 ± 15 130 ± 22 62 ± 15	82 ± 15 125 ± 20 60 ± 14	0.229 0.270 0.290
Heart rate (beat per minute; mean ± SD)	84 ± 20	86 ± 20	0.753
Respiratory Rate (cycle/min; mean ± SD)	21 ± 5	21 ± 5	0.843
Inspired fraction of oxygen (%; mean ± SD)	36 ± 11	37 ± 12	0.664
Tidal volume (mL; mean ± SD)	453 ± 106	445 ± 87	0.469
Positive end-expiratory pressure (mmHg; mean ± SD)	7.2 ± 2	7.4 ± 1	0.299
Serum albumin (g/L; mean ± SD)	24 ± 4	23 ± 5	0.257
Hemoglobin (g/dL; mean ± SD)	9.1 ± 1.2	9.2 ± 1.1	0.920
Blood urea nitrogen (mmol/L; mean ± SD)	20.7 ± 9.7	16.7 ± 8.4	0.017
Ultrafiltration (Liters) Prescribed Performed	1.88 ± 0.9 1.83 ± 0.9	1.81 ± 0.9 1.70 ± 0.9	0.374 0.337

## Data Availability

The data that support the findings of this study are available from S.F. but restrictions apply to the availability of these data, which were used under license for the current study, and so are not publicly available. Data are, however, available from the authors upon reasonable request and with permission of S.F.
